# Structural identification of catalytic His158 of PtMAC2p from *Pseudozyma tsukubaensis*, an acyltransferase involved in mannosylerythritol lipids formation

**DOI:** 10.3389/fbioe.2023.1243595

**Published:** 2023-10-18

**Authors:** Yusuke Nakamichi, Azusa Saika, Masahiro Watanabe, Tatsuya Fujii, Tomotake Morita

**Affiliations:** ^1^ Bioconversion Group, Research Institute for Sustainable Chemistry, National Institute of Advanced Industrial Science and Technology (AIST), Higashi-Hiroshima, Japan; ^2^ Biochemical Group, Research Institute for Sustainable Chemistry, National Institute of Advanced Industrial Science and Technology (AIST), Tsukuba, Japan

**Keywords:** crystal structure, mannosylerythritol lipids, acyltransferase, *Pseudozyma tsukubaensis*, PtMAC2p

## Abstract

Mannosylerythritol lipids (MELs) are extracellular glycolipids produced by the basidiomycetous yeast strains. MELs consist of the disaccharide mannosylerythritol, which is acylated with fatty acids and acetylated at the mannose moiety. In the MEL biosynthesis pathway, an acyltransferase from *Pseudozyma tsukubaensis*, PtMAC2p, a known excellent MEL producer, has been identified to catalyze the acyl-transfer of fatty acid to the C3′-hydroxyl group of mono-acylated MEL; however, its structure remains unclear. Here, we performed X-ray crystallography of recombinant PtMAC2p produced in *Escherichia coli* and homogeneously purified it with catalytic activity *in vitro*. The crystal structure of PtMAC2p was determined by single-wavelength anomalous dispersion using iodide ions. The crystal structure shows that PtMAC2p possesses a large putative catalytic tunnel at the center of the molecule. The structural comparison demonstrated that PtMAC2p is homologous to BAHD acyltransferases, although its amino acid-sequence identity was low (<15%). Interestingly, the HXXXD motif, which is a conserved catalytic motif in the BAHD acyltransferase superfamily, is partially conserved as His158-Thr159-Leu160-Asn161-Gly162 in PtMAC2p, *i.e.*, D in the HXXXD motif is replaced by G in PtMAC2p. Site-directed mutagenesis of His158 to Ala resulted in more than 1,000-fold decrease in the catalytic activity of PtMAC2p. These findings suggested that His158 in PtMAC2p is the catalytic residue. Moreover, in the putative catalytic tunnel, hydrophobic amino acid residues are concentrated near His158, suggesting that this region is a binding site for the fatty acid side chain of MEL (acyl acceptor) and/or acyl-coenzyme A (acyl donor). To our knowledge, this is the first study to provide structural insight into the catalytic activity of an enzyme involved in MEL biosynthesis.

## 1 Introduction

**FIGURE 1:**
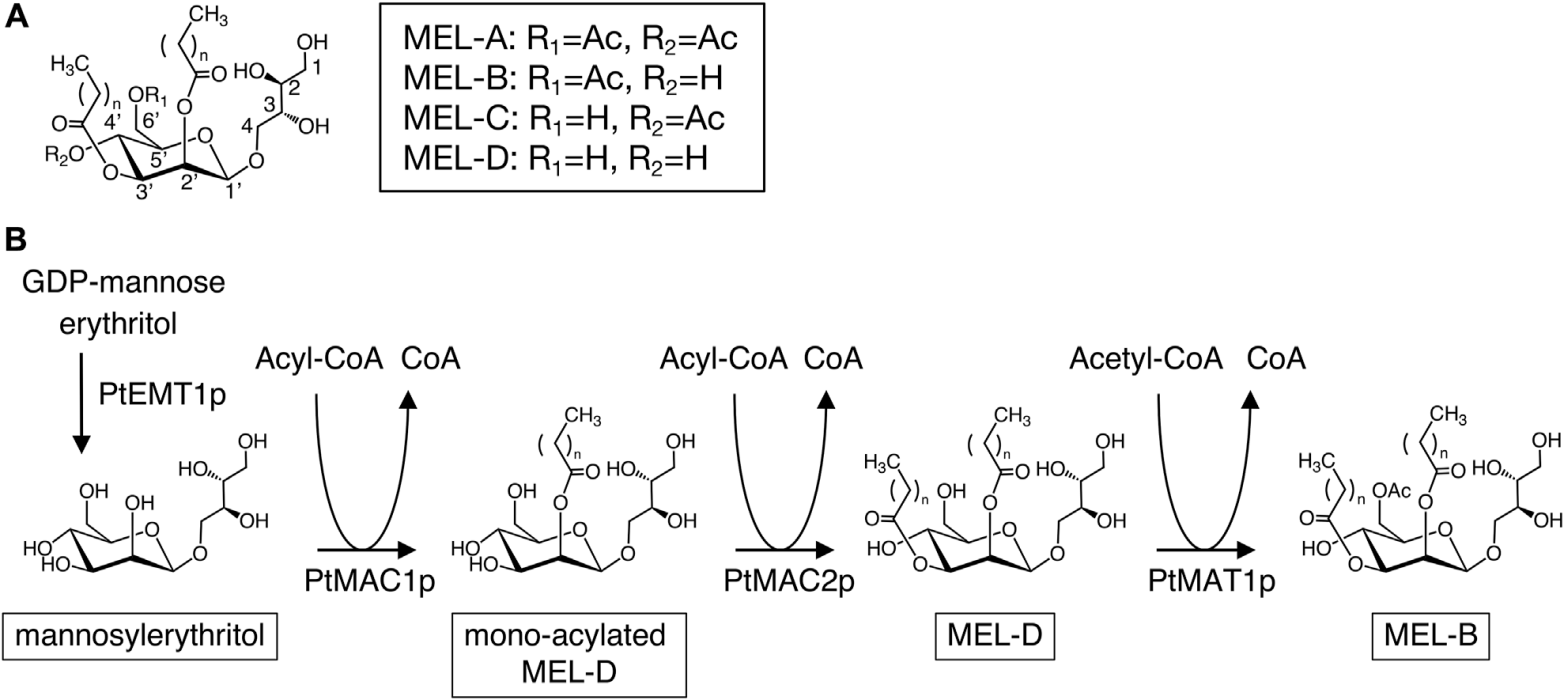
**(A)** Molecular structure of conventional MELs. **(B)** Biosynthetic pathway of MELs in *P. tsukubaensis*.

Mannosylerythritol lipids (MELs) are biosurfactants produced by basidiomycetous yeast strains, which have unique properties such as damaged skin restoration, increased DNA transfection efficiency in liposome systems, and antibacterial, anticancer, and antioxidative activities (Kitamoto et al., 1993; Shu et al., 2019; Coelho et al., 2020; Bakur et al., 2022; Kondo et al., 2022). MELs are composed of two parts: a hydrophilic 4-*O*-β-D-mannopyranosyl-D-erythritol moiety and a hydrophobic moiety containing fatty acyl chains (C4–C18) at the C2′ and C3′ positions of the mannose moiety. MELs also possess one or two acetyl groups at C4′ and/or C6′ of the mannose moiety. MELs are categorized as MEL-A (acetylated at C4′ and C6′ position), -B (acetylated at C6′ position), -C (acetylated at C4’ position), and -D (deacetylated) based on their acetylated positions (Figure 1A) (Kitamoto et al., 1990; Hewald et al., 2006).

*Pseudozyma tsukubaensis* is known as an excellent producer of diastereomeric MEL-B which contain 4-*O*-β-D-mannopyranosyl-(2*R*,3*S*)-erythritol (*R*-form) (Fukuoka et al., 2008; Morita et al., 2010), while conventional types of MELs contain 4-*O*-β-D-mannopyranosyl-(2*S*,3*R*)-erythritol (*S*-form) (Figure 1A). MEL-B biosynthesis in *P. tsukubaensis* is carried out mainly by four enzymes: the glycosyltransferase PtEMT1p, two acyltransferases, PtMAC1p and PtMAC2p and acetyltransferase PtMAT1p (Figure 1B) (Saika et al., 2016). Initially, mannosylerythritol is produced from GDP-mannose and erythritol using PtEMT1p; then, PtMAC1p and PtMAC2p attach the fatty acid side chain at position C2′ and C3′of mannosylerythritol, resulting in a di-acylated MEL (MEL-D) (Saika et al., 2016). Subsequently, PtMAT1p catalyzes acetylation at C6′ positions of a MEL-D, resulting in mature MEL-B (Figure 1B). Thus, the catalytic properties of enzymes involved in MEL biosynthesis are key factors in determining the structures of MEL products. A deletion mutant of the gene encoding PtMAC2p in *P. tsukubaensis*, which is strain Δ*PtMAC2*, leads to the accumulation of MEL acylated at the C2′ position of the mannose moiety (mono-acylated MEL-D in Figure 1B), indicating that PtMAC2p catalyzes acylation at the C3′ position of MEL (Saika et al., 2018). However, the structures of these enzymes have not been investigated.

Therefore, in the present study, we expressed and purified recombinant PtMAC2p. Subsequently, X-ray crystallography of PtMAC2p was performed to elucidate the catalytic mechanism. Structural comparison of PtMAC2p with structurally homologous proteins, sequence analysis, and site-directed mutagenesis provided insights into the catalytic reaction, including the putative binding states of the substrates.

## 2 Materials and methods

### 2.1 Construction of plasmids

The *PtMAC2* fragment (NCBI accession No., LC768982; 1.7 kb) was amplified via PCR using the cDNA of *P. tsukubaensis* NBRC1940 as a template and the following set of oligonucleotide primers: 5′-CGC​GCG​GCA​GCC​ATA​TGC​TAG​GAG​ATC​AAG​TTT​GGA​AGG​AG-3′ (forward) and 5′-GTT​AGC​AGC​CGG​ATC​CTC​GAG​CTA​AAG​CTT​GGC​CTC​AGG​AG-3′ (reverse). We inserted the 1.7-kb *PtMAC2* fragment into *Nde*I- and *Xho*I-digested pET15b using an In-Fusion Cloning Kit (TaKaRa Bio, Shiga, Japan) according to the manufacturer’s instructions, which yielded pET-NBRCMAC2. The ligated gene fragments were verified by DNA sequencing. The plasmid pET-NBRCMAC2_H158A for mutant PtMAC2p (H158A) expression was constructed by PCR using pET-NBRCMAC2 as a template, KOD one (Toyobo, Osaka, Japan) as polymerase, and the following set of oligonucleotide primers:5′-GGTCATGAATGCTTCTGCTACGCTAAATGGTCACCGCATG-3’ (forward) and 5′-CAT​GCG​GTG​ACC​ATT​TAG​CGTAGCAGA​AGC​ATT​CAT​GAC​C-3’ (reverse), including a mutation site (under lines). PCR conditions were as follows: 98°C for 10 s, 54°C for 5 s, and 68°C for 30 s with a total 30 cycles. The PCR mixture was then treated by *Dpn*I at 37°C for 1 h. Subsequently, the PCR product was transformed into DH5α competent cells (Toyobo). The plasmid pET-NBRCMAC2_G162D for mutant PtMAC2p (G162D) expression was also constructed by PCR using pET-NBRCMAC2 as a template, KOD one (Toyobo, Osaka, Japan) as polymerase, and the following set of oligonucleotide primers:5′-CTAAATGATCACCGCATGCTCTTCCAAGGTTC-3’ (forward) and 5′-GCG​GTGATCATT​TAG​CGT​ATG​AGA​AGC​ATT​CAT​GAC​CA-3’ (reverse), including a mutation site (under lines). PCR conditions were as follows: 98°C for 10 s, 54°C for 5 s, and 68°C for 30 s with a total 30 cycles. The PCR mixture was then ligated using an In-Fusion Cloning Kit (TaKaRa Bio) according to the manufacturer’s instructions. The resultant plasmids pET-NBRCMAC2_H158A and pET-NBRCMAC2_G162D were amplified in DH5α cells and extracted using QIAprep Miniprep kit (QIAGEN, Venlo, Netherlands) according to the manufacturer’s instructions. The mutation was verified by DNA sequencing.

### 2.2 Protein expression and purification

*Escherichia coli* BL21(DE3) cells harboring pET-NBRCMAC2, pET-NBRCMAC2_H158A, and pET-NBRCMAC2_G162D were cultured respectively at 37°C in 1 L of LB medium (Nacalai Tesque, Kyoto, Japan) supplemented with 100 μg mL^−1^ sodium ampicillin in 3-L Erlenmeyer Flasks with baffles. When the turbidity (O.D. value) at 600 nm reached 0.4–0.6, the cells were cooled with ice water for 10 min and isopropyl d-thiogalactopyranoside (IPTG) was added to the culture to a final concentration of 0.1 mM. The cells were then cultured at 18 °C for 20 h. Subsequently, the cells were collected by centrifugation at 6,000 × *g* for 10 min at 4°C and washed with phosphate-buffered saline (PBS), which is composed of 137 mM NaCl, 8.1 mM Na_2_HPO_4_, 2.68 mM KCl, and 14.7 mM KH_2_PO_4_, at pH 7.2. The cells were then resuspended in buffer A (PBS supplemented with 150 mM NaCl). The cells were ultrasonically disrupted in ice water using an ultrasonic disruptor UD-211 (Tomy, Tokyo, Japan). The supernatant was obtained by centrifugation at 30,000 × *g* for 30 min at 4°C.

PtMAC2p purification from the supernatants was achieved through a 0.22-μm polyethersulfone membrane. The filtered samples were applied to a HisTrap HP column (5 mL; Cytiva, Tokyo, Japan) that had been equilibrated with Buffer A. The HisTrap HP column was washed with 40 mL of buffer A supplemented with 10 mM imidazole and 40 mL of buffer A with 40 mM imidazole. PtMAC2p was eluted with 20 mL of buffer A containing 300 mM imidazole. The sample was desalted and equilibrated with a buffer containing 20 mM potassium phosphate (KPi) at pH 7.2 by ultrafiltration using Vivaspin 20-5K (Sartorius, Göttingen, Germany). Subsequently, the sample was applied to a HiTrap Q HP anion exchange column (5 mL; Cytiva) that had been equilibrated with the same buffer. The HiTrap Q HP column was washed with 20 mL of 20 mM KPi (pH 7.2) supplemented with 100 mM NaCl and PtMAC2p was eluted with 15 mL of 20 mM KPi (pH 7.2) with 200 mM NaCl. PtMAC2p H158A was purified in the same manner as the wild-type enzyme. The concentration of the purified PtMAC2p was determined by measuring the absorbance at 280 nm. The extinction coefficient at 280 nm was calculated based on the amino acid sequence 58,330 M^–1^ cm^–1^ (*A*_280_ was 0.964 when 1 mg mL^–1^ of PtMAC2p was in solution).

### 2.3 Enzyme assay

PtMAC2p activity was assayed using the 5,5-dithio-bis-(2-nitrobenzoic acid) reagent (Ellman’s reagent). The assay mixture containing 50 mM sodium phosphate buffer (pH 7.2), 0.1 mM lauroyl-CoA, 1 mM mono-acylated MEL-D produced from *P. tsukubaensis* strain Δ*PtMAC2* (Saika et al., 2018), 1 mM DTNB, and PtMAC2p (0.2 µg of wild-type enzyme, 2 µg of H158A, or 7 µg of G162D) was incubated at 25°C. The increase in the reaction product was measured by the increase of absorbance at 412 nm of 2-nitro-5-thiobenzoic acid, the reduced form of DTNB. The product concentration was calculated using a molar absorption coefficient at 412 nm as 13,600 M^–1^ cm^–1^. All assays were performed in triplicate.

### 2.4 X-ray crystallography

For crystallization, PtMAC2p was concentrated to 5 mg mL^–1^ by ultrafiltration using a Vivaspin 20-5K. PtMAC2p crystals were grown at 15°C using hanging drop vapor diffusion. The purified PtMAC2p solution was mixed with equal volume of reservoir solution 1, containing 19% polyethylene glycol (PEG) 3350 and 200 mM lithium acetate. Also, a PtMAC2p solution supplemented with 5% glycerol was mixed with equal volume of reservoir solution 2 containing 21% PEG 3350 and 300 mM lithium chloride. Crystals were grown at 15°C for a week. The crystals prepared from reservoir solutions 1 and 2 were designated as types A and B, respectively. Native crystals types A and B were soaked in the reservoir solutions supplemented with 25% and 20% glycerol as cryoprotectants, respectively, and then flash cooled in liquid nitrogen. For phasing, type A crystals were soaked in reservoir solution 1 supplemented with 1 M potassium iodide (KI) for a few seconds and then flash-cooled in liquid nitrogen.

All diffraction data were collected at the BL44XU station at SPring-8 (Hyogo, Japan). Native datasets were collected at λ = 0.9 Å, while an anomalous dataset of KI-derivative crystal was collected at λ = 1.6 Å. All the datasets were processed and scaled using XDS version 10 Jan 2022 (Kabsch, 2010). The initial phase was solved by single anomalous dispersion (SAD) using the KI-derivative dataset with Autosol from Phenix 1.20.1 (Terwilliger et al., 2009; Liebschner et al., 2019). Subsequently, the model was manually completed using Coot (Emsley et al., 2010) and refined using Phenix.refine (Afonine et al., 2012). The obtained model was used as a search model for the native datasets. The phases of the native crystals were solved by molecular replacement using the structure of KI-derivative crystal as a search model with Phaser (McCoy et al., 2007). The coordinates were refined using Phenix.refine. After each refinement cycle, the models were manually adjusted using Coot software. Structural models were generated using Pymol 2.5.0 (Schrödinger, LLC). The quality of the refined model was verified using MolProbity 4.5.2 (Williams et al., 2017). The root mean square deviations (RMSD) value of Cα-atoms between structures in crystals types A and B was calculated using LSQKAB (Kabsch, 1976). The volume of the protein cavity was calculated by CASTp (Tian et al., 2018). The atomic coordinates and structure factors were deposited in the Protein Data Bank (PDB) under accession codes 8JOR (type A crystal) and 8JOS (type B crystal).

## 3 Results

### 3.1 Overall structure

Recombinant PtMAC2p with a polyhistidine tag at the N-terminus was expressed in *E*. *coli* and was homogeneously purified (Supplementary Figure S1). Purified PtMAC2p catalyzes the transfer of a fatty acid of lauroyl-CoA (C12) to mono-acylated MEL-D *in vitro*. Two types of PtMAC2p crystals (A and B) were obtained using the purified enzyme (Supplementary Figure S2). Both crystals belong to the orthorhombic space group *P*2_1_2_1_2_1_, while their *b* axes differ by about 10 Å (Table 1).

**TABLE 1:** Statistics for X-ray crystallography.

	KI-derivative	Type A	Type B
Diffraction source	BL44XU, SPring-8	BL44XU, SPring-8	BL44XU, SPring-8
Wavelength (Å)	1.6	0.9	0.9
Temperature (K)	100	100	100
Detector	Eiger X 16M	Eiger X 16M	Eiger X 16M
Crystal-detector distance (mm)	160	200	250
Rotation range per image (°)	0.1	0.1	0.1
Total rotation range (°)	360	200	200
Exposure time per image (s)	0.1	0.1	0.1
Space group	*P*2_1_2_1_2_1_	*P*2_1_2_1_2_1_	*P*2_1_2_1_2_1_
*a*, *b*, *c* (Å)	53.84, 82.89, 132.43	53.04, 83.07, 131.04	53.91, 71.91, 128.65
Resolution range (Å)	45.15–2.90 (3.00–2.90)^a^	44.73–1.45 (1.50–1.45)	47.96–3.42 (1.64–1.59)
Total No. of reflections	169,444 (17,527)	764,963 (76,535)	505,781 (49,594)
No. of unique reflections	13,487 (1,347)	103,745 (10,118)	68,145 (6,607)
Completeness (%)	98.4 (100)	99.8 (98.9)	99.8 (98.5)
Redundancy	12.6 (13.0)	7.4 (7.6)	7.4 (7.5)
⟨*I*/σ(*I*)⟩	13.3 (1.6)	15.7 (2.0)	18.7 (1.2)
CC_1/2_	0.996 (0.768)	0.999 (0.666)	1.000 (0.612)
*R*_p.i.m_	0.052 (0.626)	0.030 (0.513)	0.020 (0.581)
Overall *B* factor from Wilson plot (Å^2^)	79.0	17.5	27.1
Resolution range (Å)	—	35.08–1.45	47.94–1.59
Completeness (%)	—	99.8	99.8
σ cut-off	—	0	0
No. of reflections, working set	—	103,733	68,133
No. of reflections, test set	—	5,187	3,407
Final *R*_cryst_	—	17.1	18.8
Final *R*_free_	—	19.4	21.3
No. of non-H atoms	—	—	—
Protein	—	4,498	4,344
Ligand	—	16	20
chloride ion	—	–	1
Water	—	702	223
Total	—	5,216	4,588
R.m.s deviations	—	—	—
Bonds (Å)	—	0.006	0.007
Angles (°)	—	0.897	0.969
Average *B* factors (Å^2^)	—		
Protein	—	20.3	33.5
Ligand	—	39.0	50.1
chloride ion	—	—	35.2
Water	—	30.9	35.6
Ramachandran plot	—		
Most favoured (%)	—	98.2	97.4
Allowed (%)	—	1.8	2.6

^a^ Values for the outer shell are provided in parentheses.

**FIGURE 2:**
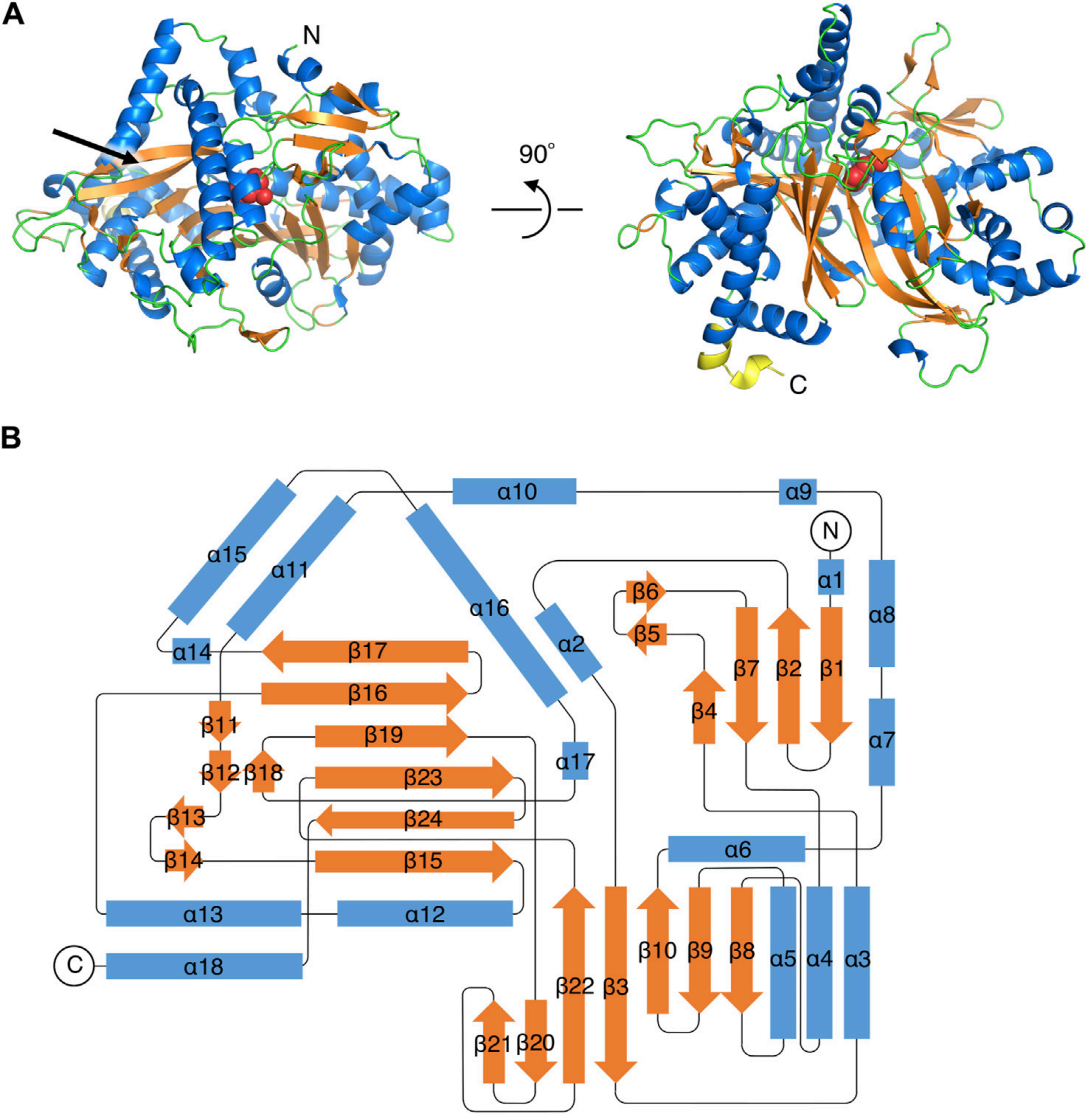
Overall structure of PtMAC2p. **(A)** A ribbon model of PtMAC2p structure in type B crystal. Blue, orange, and green indicate helices, strands, and loops, respectively. The yellow region (residues 540–549) at the C-terminal is disordered in the type A crystal. Red spheres indicate His158. “N” and “C” indicate N- and C-terminus, respectively. **(B)** A topology diagram of the PtMAC2p structure.

**FIGURE 3:**
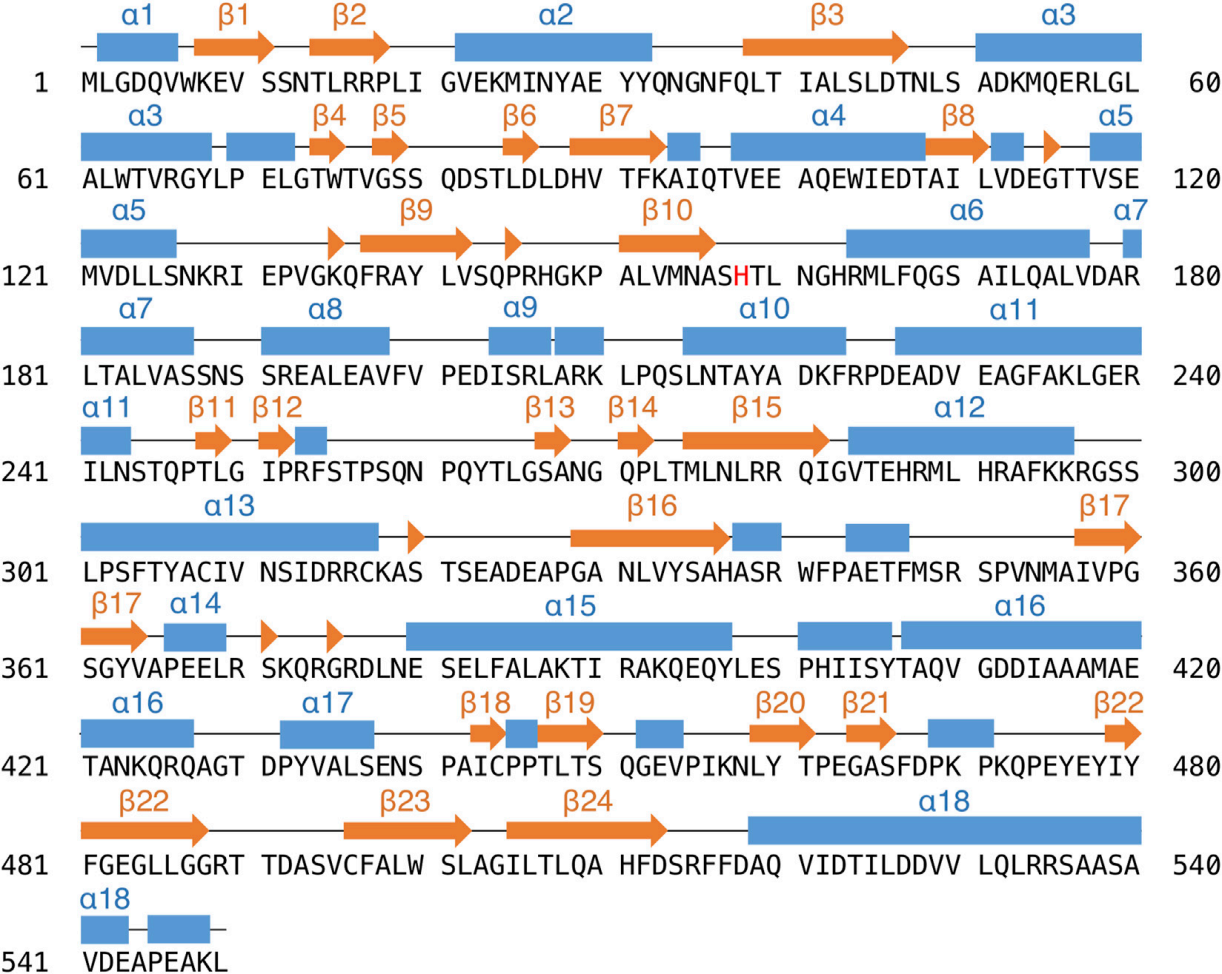
The amino acid sequence and secondary structures of PtMAC2p. Strands and helices are indicated by orange arrows and blue boxes.

The initial structure of PtMAC2p was determined by the SAD-phase method using a type A KI-derivative crystal (Table 1; Supplementary Figure S3). Native crystals in types A and B were diffracted to 1.45 and 1.59 Å resolutions, respectively (Table 1). PtMAC2p is a monomer and forms α/β-folds which are composed of 18 α-helices (α1–α18) and 24 β-strands (β1–β24) (Figures 2, 3). In the type A crystal, residues Met1 to Ser539 and residues numbered −7 to 0 (GLVPRGSH), which are from the DNA sequence included in the pET15b vector, were modeled using a 2*F*o-*F*c map at 1.0 σ (Figure 3). In contrast, Met1 to Leu549, Leu549 is the C-terminal residue of PtMAC2p, were modeled in crystal type B (Figure 2A; Figure 3). Residues 540–549 can be modeled in crystal type B because these residues interacted with a symmetric molecule in the crystal owing to crystal packing, although these residues in type A were disordered, suggesting that the C-terminus of PtMAC2p is a flexible region (the yellow region in Figure 2A). The RMSD value of 539 Cα atoms (residues 1–539) between the two PtMAC2p models was 0.643 Å, indicating that the overall structures were almost identical. PtMAC2p possesses a cleft at the center of the molecule (indicated by the arrow in Figure 2A), suggesting that this cleft is a putative active site.

Proteins structurally homologous to PtMAC2p were searched using the DALI server (Holm, 2020). This search revealed that trichothecene 15-*O*-acetyltransferase (TRI3), which belongs to the BAHD acyltransferase superfamily, showed the highest similarity to PtMAC2p with a *Z*-score of 29.4, despite the amino acid-sequence identity between PtMAC2p and TRI3 being low (<15%) (Table 2) (Garvey et al., 2009). Other homologous proteins had relatively low *Z*-scores (<22.7).

**TABLE 2:** Closest structural matches to PtMAC2p calculated using the Dali server. The top 10 matches based upon Dali *Z*-score (excluding duplicates) are shown.

Proteins	Organisms	Identity (%)	Z-score	RMSD (# of residues)	PDB codes
Trichothecene 15-*O*-acetyltransferase (TRI3)	*Fusarium sporotrichioides*	15	29.4	2.8 Å (448)	3fp0
Polyketide synthase associated protein 5 (nonribosomal peptide synthetase)	*Mycobacterium tuberculosis*	12	22.7	3.3 Å (354)	1q9j
Nonribosomal peptide synthetase PchE	*Pseudomonas aeruginosa* PAO1	13	21.4	3.4 Å (375)	7en2
Nonribosomal peptide synthetase SgcC5	*Streptomyces globisporus*	13	20.5	3.7 Å (379)	4znm
Nonribosomal peptide synthetase ObiF1	*Burkholderia diffusa*	10	20.1	3.6 Å (382)	6n8e
Nonribosomal peptide synthetase AmbE	*Pseudomonas aeruginosa* PAO1	10	19.9	3.9 Å (372)	7r9x
Nonribosomal peptide synthetase IgrA	*Brevibacillus parabrevis*	11	17.9	3.9 Å (383)	6mfz
Nonribosomal peptide synthetase TlmII	*Streptoalloteichus hindustanus*	12	17.8	3.8 Å (354)	4hvm
Diacylglycerol *O*-acyltransferase	*Marinobacter nauticus* VT8	8	17.7	4.3 Å (331)	6chj
Coumarin synthase	*Arabidopsis thaliana*	10	16.9	3.7 Å (344)	8dqo

### 3.2 Putative active site

**FIGURE 4:**
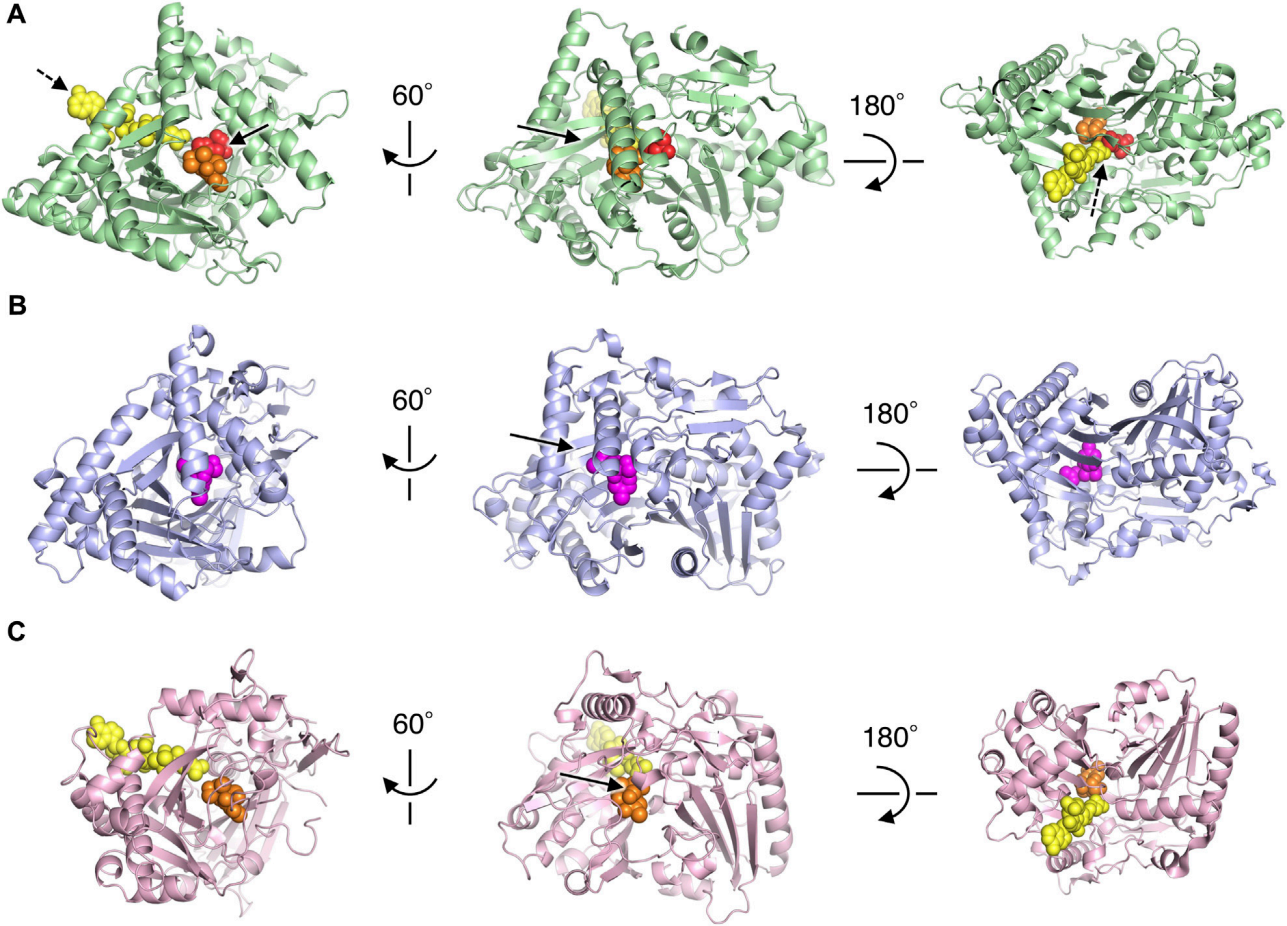
Structural comparison of PtMAC2p with TRI3 and TRI101. **(A)** PtMAC2p (green) with deoxynivalenol (orange) and CoA (yellow). Models of deoxynivalenol and CoA are from the crystal structure of TRI101 (PDB ID: 3b2s). Red spheres indicate His158. Solid and dashed arrows indicate putative binding sites of an acyl donor and acceptor, respectively. **(B)** TRI3 (light blue) complexed with 15-decalonectrin (magenta) (PDB ID: 3fp0). **(C)** TRI01 (pink) complexed with deoxynivalenol (orange) and CoA (yellow) (PDB ID: 3b2s). Deoxynivalenol and 15-decalonectrin are acyl-acceptors, and CoA is an analogue of an acyl donor.

To investigate the active site of PtMAC2p, we tried to obtain the PtMAC2 crystal with substrates, but could not. Thus, we compared its structure with a BAHD acyltransferases, TRI3, which has the highest structural similarity to PtMAC2p. The PtMAC2p model was also compared with trichothecene 3-*O*-acetyltransferase (TRI101) because the crystal structure of TRI101 in complex with an acyl donor (deoxynivalenol) and an acyl-acceptor analog (CoA) has been determined (Garvey et al., 2008) (Figure 4; Supplementary Figure S4).

**FIGURE 5:**
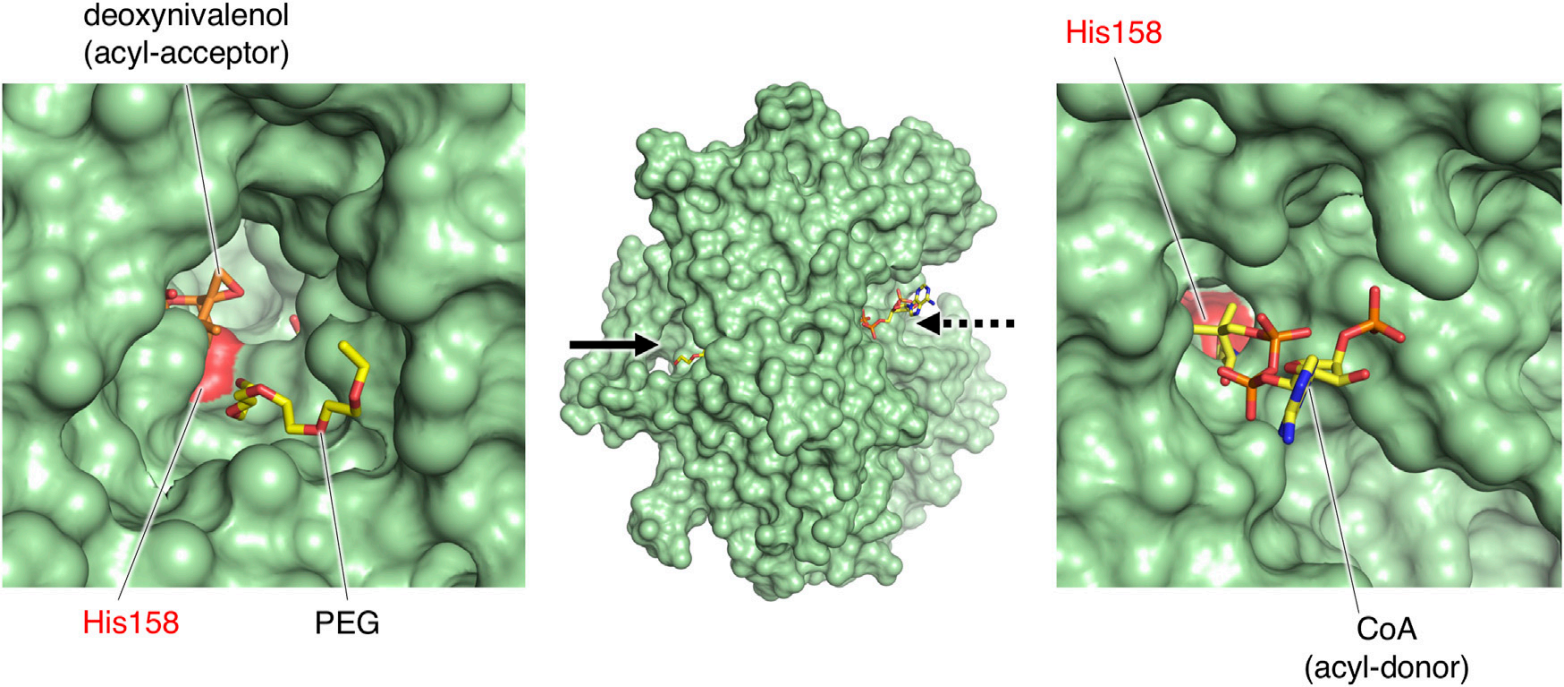
The catalytic tunnel of PtMAC2p. PtMAC2p is shown as surface model. PEG was found in the type 2 crystal of PtMAC2p. Stick models of deoxynivalenol and CoA were derived from the crystal structure of TRI101 (PDB ID:3b2s).

Structural comparison of the three enzymes indicated that the large cleft found in PtMAC2p is the binding site for an acyl acceptor (the width of the cleft entrance, 12–18 Å; the volume of the cavity, 1533 Å^3^; solid arrows in Figure 2A; Figure 4A). The acyl acceptor binding site is closed in TRI3 (the volume of the cavity, 372 Å^3^; solid arrows in Figure 4B) or remarkably narrow in TRI101 (the width of the cleft entrance, 5.6–7.2 Å; the volume of the cavity, 549 Å^3^; solid arrows in Figure 4C), suggesting that PtMAC2p has a uniquely large and open cleft that allows the binding of large substrates and products with fatty acids (*e.g.*, a substrate mono-acylated MEL and a product di-acylated MEL). In contrast, a structural comparison of PtMAC2p and TRI101 showed that the CoA residue of acyl CoA (acyl donor) binds to a site different from the cleft (dashed arrows in Figure 4A). This acyl donor binding site and the cleft (acyl acceptor binding site) are connected, resulting in a catalytic tunnel structure (Figure 5).

### 3.3 Identification of a catalytic residue

**FIGURE 6:**
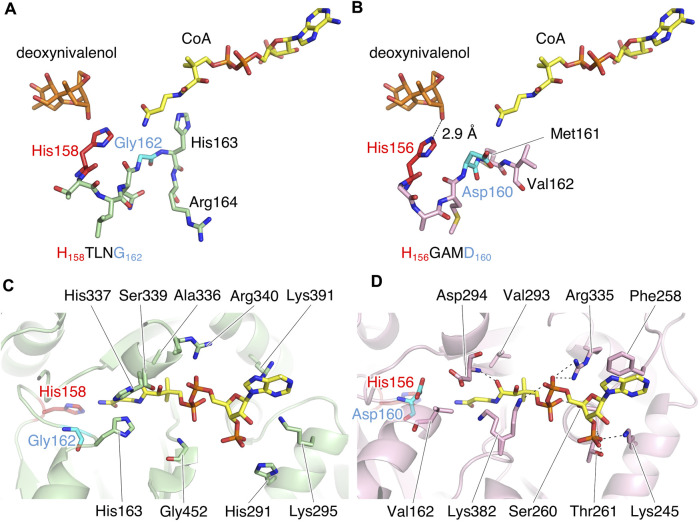
The putative active site of PtMAC2p. **(A, C)** PtMAC2p (green) with deoxynivalenol (orange) and CoA (yellow). Models of deoxynivalenol and CoA are from the crystal structure of TRI101 (PDB ID: 3b2s). **(B, D)** TRI01 (pink) complexed with deoxynivalenol (orange) and CoA (yellow) (PDB ID: 3b2s). Dashed lines indicate hydrogen-bonding interaction.

**FIGURE 7:**
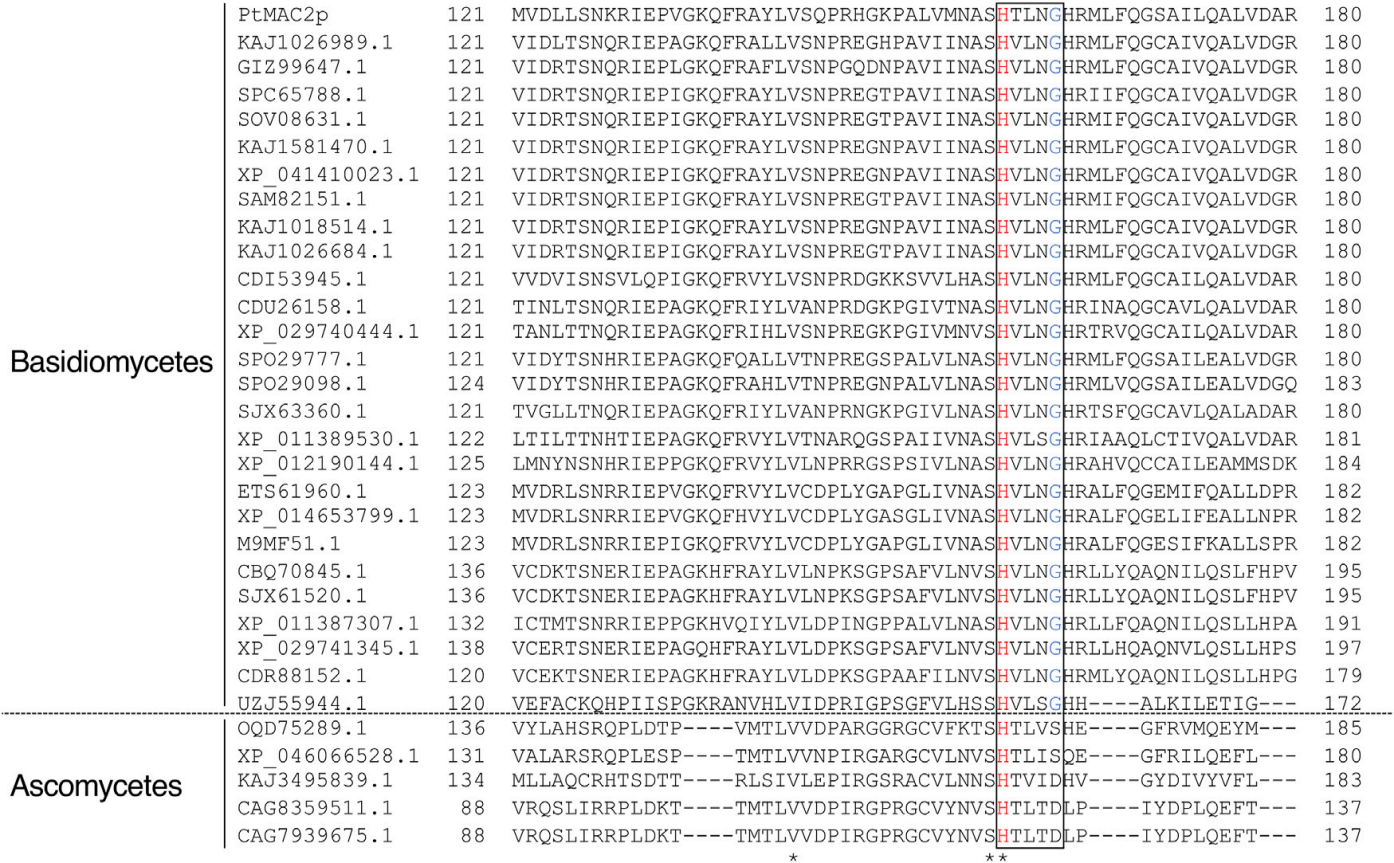
Multiple sequence alignment of PtMAC2p and its homologs. Amino acid sequences of Mac2p homologs with more than 30% identity were obtained from the protein BLAST (https://blast.ncbi.nlm.nih.gov/Blast.cgi) using the amino acid sequence of PtMAC2p. The amino acid sequences of the Mac2p homologs from the following strains were used for alignment: *Ustilago loliicola* (NCBI accession no. KAJ1026989.1), *Ustilago shanxiensis* (GIZ99647.1), *Ustilago* spp. UG-2017b (SPC65788.1) and UG-2017a (SOV08631.1), *U. hordei* (KAJ1581470.1 and XP_041410023.1), *Ustilago bromivora* (SAM82151.1), *Ustilago nuda* (KAJ1018514.1), *Ustilago tritici* (KAJ1026684.1), *Melanopsichium pennsylvanicum* 4 (CDI53945.1), *Sporisorium scitamineum* (CDU26158.1), *Sporisorium graminicola* (*Pseudozyma graminicola*) (XP_029740444.1 and XP_029741345.1), *Ustilago trichophora* (SPO29777.1 and SPO29098.1), *Sporisorium reilianum* f. sp. reilianum (SJX63360.1 and SJX61520.1), *U. maydis* 521 (XP_011389530.1 and XP_011387307.1), *Pseudozyma hubeiensis* SY62 (XP_012190144.1), *M. aphidis* (ETS61960.1), *Moesziomyces antarcticus* (XP_014653799.1), *M. antarcticus* T-34 (M9MF51.1), *S. reilianum* SRZ2 (CBQ70845.1), *S. scitamineum* (CDR88152.1), *Exobasidium rhododendri* (UZJ55944.1), *Penicillium decumbens* (OQD75289.1), *Talaromyces proteolyticus* (XP_046066528.1), *Lecanicillium saksenae* (KAJ3495839.1), and *Penicillium salamii* (CAG8359511.1 and CAG7939675.1).

TRI3 and TRI101 contain the HXXXD motif, which is essential for the catalytic activity of enzymes in the BAHD acyltransferase superfamily (Molina and Kosma, 2014). The His residue in the motif is a critical catalytic residue responsible for the deprotonation of the acyl acceptor substrate, creating a nucleophile that attacks the carbonyl carbon of the acyl CoA substrate, and resulting in the release of CoASH and the formation of ester products (Suzuki et al., 2003; Lallemand et al., 2012; Walker et al., 2013). Notably, PtMAC2p possesses His158 at the same location as His in the HXXXD motifs of TRI3 and TRI101 (Figures 6A, B). We then evaluated the sequence conservation of the motif in Mac2p homologs, which were obtained from the protein BLAST, using the amino acid sequence of PtMAC2p. Among the Mac2p homologs, His was completely conserved in the motif, but Asp was not (Figure 7). Therefore, HXXXD is incompletely conserved in PtMAC2p as His158-Thr159-Leu160-Asn161-Gly162. This strongly suggests that His158 is the catalytic residue of PtMAC2p, although the Asp residue in the motif is replaced by Gly162.

To further investigate the role of His158 in the catalytic activity, site-directed mutagenesis was performed. Substitution of His158 in PtMAC2p with Ala (H158A) resulted in more than 1000-fold decrease in catalytic activity of PtMAC2p (from 152 ± 3 μmol min^–1^ mg^–1^ in wild-type to 0.0864 ± 0.0091 μmol min^–1^ mg^–1^ in H158A mutant). This result reveals that His158 is an important catalytic residue in PtMAC2p and corresponds to the catalytic His in BAHD acyltransferases, such as TRI3 and TRI101.

### 3.4 Substrate binding site

**FIGURE 8:**
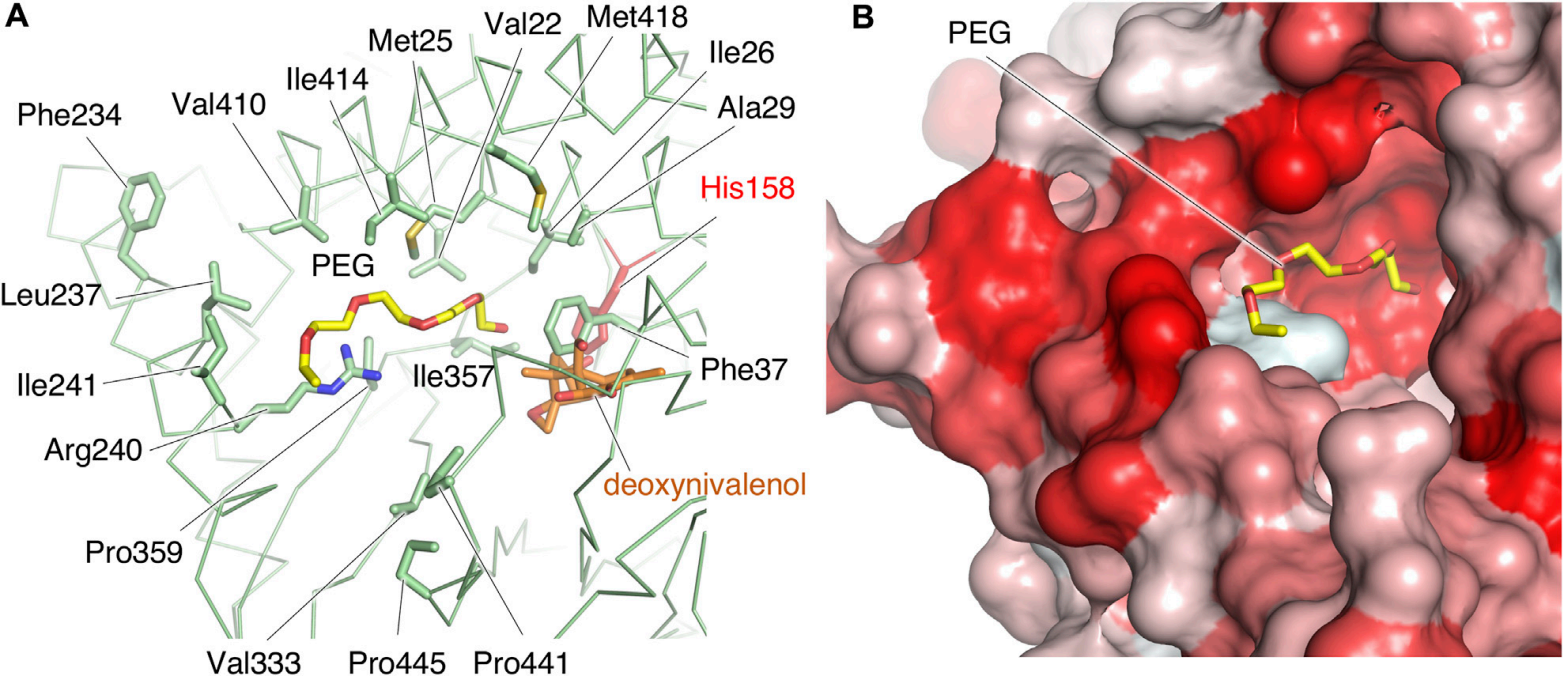
The structure of the putative binding site of acyl groups. **(A)** Hydrophobic residues and Arg240 in the cleft are shown by sticks. Deoxynivalenol model is from the crystal structure of TRI101 (PDB ID: 3b2s). **(B)** The extent of the surface hydrophobicity of PtMAC2p was calculated and represented by using the color_h script of the PyMOL software. The color is based on the hydrophobicity, with a gradient from white to red (the darker the red, the more hydrophobic the surface).

We further explored structural features related to substrate recognition. In the type A crystal, electron density map of a PEG molecule in the reservoir solution for crystallization is observed. The PEG is bound near His158 in the cleft of PtMAC2p (Figure 5; Figure 8A). Hydrophobic amino acid residues, mainly from α2 (Val22, Met25, Ile26, and Ala29) and α11 (Phe234, Leu237, and Ile241) helices, are concentrated around PEG, although a hydrophilic residue, Arg240, is also located near PEG. These residues form a hydrophobic surface in the cleft (Figure 8B). These findings suggest that the PEG mimics the binding position of the acyl group of mono-acylated MEL and/or acyl CoA and that this hydrophobic cleft may accommodate one or two acyl groups.

In contrast, a CoA residue is likely recognized on the other side of PtMAC2p (dashed arrows in Figure 4A). The putative CoA-binding site was compared to that of TRI101 (Figures 6C, D). Although the structure and amino acid residues of the CoA-binding site of TRI101 are not well conserved in PtMAC2p, the residues involved in the recognition of the CoA phosphate groups appear to be partly common. The phosphate groups of CoA interact with basic residues in TRI101, such as Lys245, Arg335, and Lys382 (Figure 6D). In PtMAC2p, His291, Lys295, Arg340, and Lys391 are candidates for electrostatic interactions with phosphate groups. In contrast, there is no residue in PtMAC2p corresponding to Phe258 in TRI101, which interacts with an adenine moiety *via* stacking. Therefore, this region in PtMAC2p is likely the CoA binding site; however, further studies are needed to identify the correct binding site for CoA.

## 4 Discussion

This is the first study providing structural insights into the catalytic reaction of an acyltransferase involved in MEL biosynthesis, PtMAC2p. The crystal structure of PtMAC2p reveals that the enzyme possesses a catalytic tunnel structure at the center of the molecule. Structural comparison of PtMAC2p and BAHD acyltransferases and site-directed mutagenesis showed that His158 is a critical catalytic residue, although the conserved catalytic motif of the BAHD acyltransferase superfamily HXXXD is incompletely conserved in PtMAC2p as His158-Thr159-Leu160-Asn161-Gly162. Structural comparison also suggested the presence of binding sites of the acyl donors and acceptors in PtMAC2p.

DALI identified a variety of enzymes with structures homologous to PtMAC2p (Table 2). In the top 10 matches, many non-ribosomal peptide synthases, which are involved in the synthesis of various bioactive natural products, ranging from therapeutic drugs (antibiotics, antitumor agents, and immunosuppressants) to virulence factors, were included (Buglino et al., 2004; Tao et al., 2007; Kreitler et al., 2019; Reimer et al., 2019; Patteson et al., 2022; Wang et al., 2022). Unexpectedly, there are only four CoA-dependent acyltransferases and acetyltransferases: TRI3, polyketide synthase-associated protein 5, diacylglycerol *O*-acyltransferase, and coumarin synthase (Petronikolou and Nair, 2018; Kim et al., 2023). These CoA-dependent enzymes share the HXXXD motif, while non-ribosomal peptide synthases preserve the HHXXXDX_14_Y motif. In PtMAC2p, the HXXXD motif is incompletely conserved, as H_158_VLNG_162_, and the first His and last Tyr residues in HHXXXDX_14_Y are not conserved in PtMAC2p, indicating that PtMAC2p is categorized as another enzyme group. In addition, the sequence HVL(N/S)G (H_158_VLNG_162_ in PtMAC2p) appears to be conserved in the Mac2p homologs of basidiomycetes, whereas the sequence is incompletely conserved in those of ascomycetes (Figure 7). In contrast, in MAC1p, such as in enzymes from *Moesziomyces antarcticus* T-34 (UniProtKB accession ID, M9LYJ5.1), *U. hordei* (CCF52716), *U. maydis*, and *M. aphidis* (ETS61961), the HXXXD motif is conserved, suggesting that MAC1p functions as a typical BAHD acyltransferase. The catalytic motif HVL(N/S)G is likely to be conserved in basidiomycetous Mac2p, but not in Mac1p or ascomycetous Mac2p.

Site-directed Asp mutagenesis in the HXXXD motif of the BAHD acyltransferase superfamily to Ala in the BAHD acyltransferases is reported to cause serious decrease in the activity (<0.3% relative to wild-types) (Suzuki et al., 2003; Bayer et al., 2004). In Ss5MaT1, malonyl-CoA:anthocyanin 5-*O*-glucoside-6‴-*O*-malonyltransferase of *Salvia splendens* flowers, the Asp residue seems to be involved in recognition of the acyl-acceptors rather than the acyl donor (Suzuki et al., 2003). Structural analysis of Dm3MaT3, a BAHD acyltransferase, also suggested that this Asp residue interacts with Arg residues and likely plays a structural, rather than a catalytic, role in enzyme function (Unno et al., 2007; Molina and Kosma, 2014). In PtMAC2p, Gly162 is probably involved in the enzyme reaction by a different mechanism from His158, since the crystal structure indicates that the Gly162 does not interact directly with both substrates and the catalytic residue, His158. In fact, substitution of Gly162 for Asp decreased enzymatic activity to 3% of wild-type enzyme (from 152 ± 3 μmol min^–1^ mg^–1^ in wild-type to 4.63 ± 0.06 μmol min^–1^ mg^–1^ in G162D mutant). The orientation of the side chain of His163, the residue next to Gly162, is markedly different from that of Met161 in TRI101 (Figures 6A, B), resulting in the side chain of His163 constituting the CoA binding site (Figure 6C). Thus, Gly162 in the motif HXXXG is likely involved in the formation of the active site in PtMAC2p. Further site-directed mutagenesis and elucidation of the complex structure of PtMAC2p with substrates will help to clarify the mechanism of substrate recognition in PtMAC2p, including the function of Gly162, in future.

**FIGURE 9:**
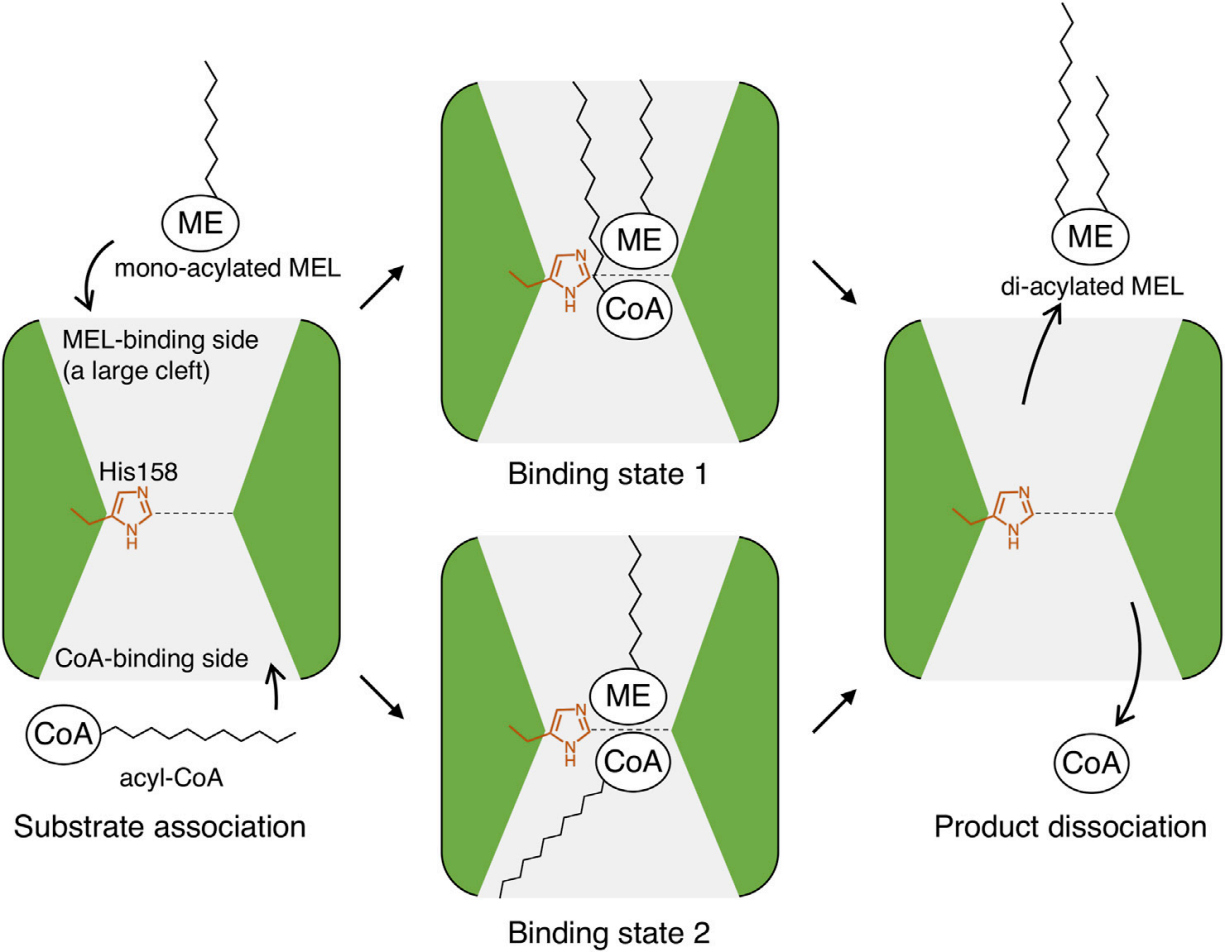
Putative association and dissociation model of substrates and products in PtMAC2p. ME, mannosylerythritol moiety; CoA, CoA residue; and zig-zag lines, alkyl chains of acyl-groups.

The association and dissociation mechanisms of the substrates and products in PtMAC2p were deduced based on the crystal structure. As shown in Figures 4, 5, a structural comparison of PtMAC2p with TRI101 indicates that the binding sites of the mono-acylated MEL and CoA residues are different. Thus, mono-acylated MEL and acyl CoA are considered to approach the catalytic center in opposite directions (Figure 9, left). After the enzymatic reaction, the products, di-acylated MEL and CoA, likely dissociate in different directions (Figure 9, right). Thus, two possible states of substrate-binding can be estimated. The acyl groups of both mono-acylated MEL and acyl CoA are located in the same direction in a cleft (Figure 9, binding state 1) because the cleft is large enough to accommodate the two acyl groups (Figure 5, left). Alternatively, both acyl groups could be positioned in opposite directions (Figure 9, binding state 2) because there is a cavity that can accommodate acyl groups in a location other than the large cleft (Supplementary Figure S5).

The length of the acyl group of the MEL produced by microorganisms is species-specific and depends on the substrate specificity of the enzymes (Beck et al., 2019; Becker et al., 2021). For example, MELs from *P. tsukubaensis* have mainly C10–14 acyl-groups at C3′ position (Morita et al., 2007). Also, while the MELs from *U. maydis* and *U. hordei* mainly have C12–16 acyl-groups at C3′ position (Deinzer et al., 2019), those from *M. aphidis* are on C8–C10 (Beck et al., 2019; Becker et al., 2021). Therefore, the substrate specificity and structure of the substrate-binding sites should differ. If binding state 1 in Figure 9 is applied to the substrate association in Mac2p, the fatty acid of the acyl donor (acyl CoA) binds to the large cleft, and the structure of the cleft may be responsible for the lengths of the C3′ acyl group in the produced MELs. When the hydrophobic residues in the cleft of Mac2p were compared, most residues were conserved among the Mac2p homologs (Supplementary Figure S5). Thus, it is possible that the structural determinant of the chain length of C3’ acyl-group of MEL is not located on the hydrophobic region. Further structural studies are required to elucidate the determinants Mac2p substrate specificity.

## 5 Conclusion

First, we determined the structure of PtMAC2p. The enzyme possesses a putative catalytic tunnel at the center of the molecule. Structural comparison with structurally homologous proteins revealed that the HXXXD motif, which is essential for the catalytic activity of enzymes in the BAHD acyltransferase superfamily, is incompletely conserved in PtMAC2p as His158-Thr159-Leu160-Asn161-Gly162, that is, D in the HXXXD motif is replaced by G in PtMAC2p. Notably, the alanine replacement of His158 resulted in a remarkable decrease in activity, revealing that His158 is a critical catalytic residue. The concentrated hydrophobic residues appear to be recognition sites for the acyl groups. This study is expected to improve our understanding of the mechanisms underlying MEL biosynthesis.

## Data Availability

The datasets presented in this study can be found in online repositories. The names of the repository/repositories and accession number(s) can be found in the article/Supplementary Material.
