# Supplementary material for: Structural identification of catalytic His158 of PtMAC2p from Pseudozyma tsukubaensis, an acyltransferase involved in mannosylerythritol lipids formation
Source: Front Bioeng Biotechnol. 2023 Oct 18;11:1243595. doi: 10.3389/fbioe.2023.1243595 (PMC10619693; doi:10.3389/fbioe.2023.1243595)
Supplement: Supplementary file 1 [file DataSheet1.pdf]

Supplementary Material

Structural identification of catalytic His158 of PtMAC2p from *Pseudozyma tsukubaensis*, an acyltransferase involved in mannosylerythritol lipids formation

Yusuke Nakamichi[1], Azusa Saika[2], Masahiro Watanabe[1], Tatsuya Fujii[1], and Tomotake Morita[1]*

*** Correspondence:** Tomotake Morita: morita-tomotake@aist.go.jp

1 Supplementary Figures

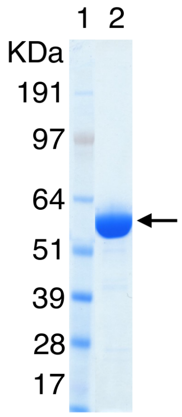

Supplementary Figure 1. SDS-PAGE profile of purified PtMAC2p. Lane 1, molecular mass standards; lane 2, purified PtMAC2p. The arrow indicates the position of PtMAC2p.

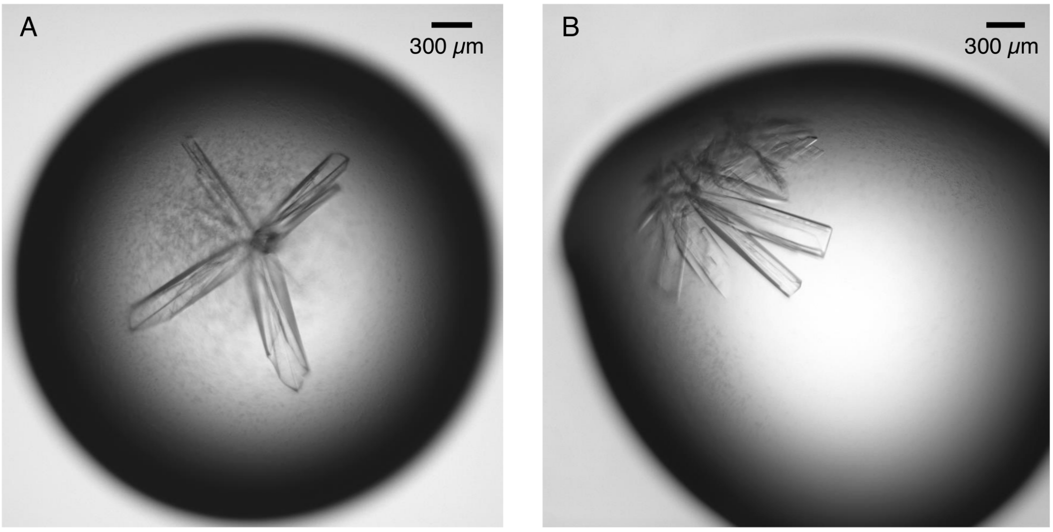

Supplementary Figure 2. Crystals of PtMAC2p in types A and B. Both crystals belong to space group $P2_12_12_1$ with different unit cell dimensions, a = 53.04 Å, b = 83.07 Å, and c = 131.04 Å (type A), and a = 53.91 Å, b = 71.91 Å, and c = 128.65 Å, respectively.

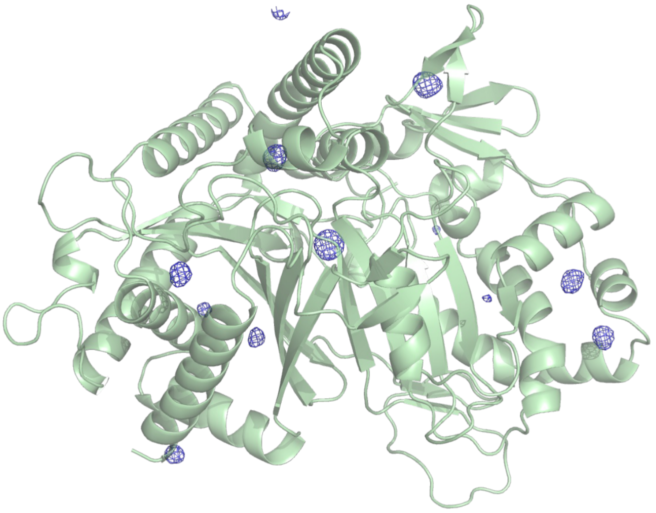

Supplementary Figure 3. Iodide peaks in anomalous difference Patterson map at 2.9 Å resolution are shown as blue mesh.

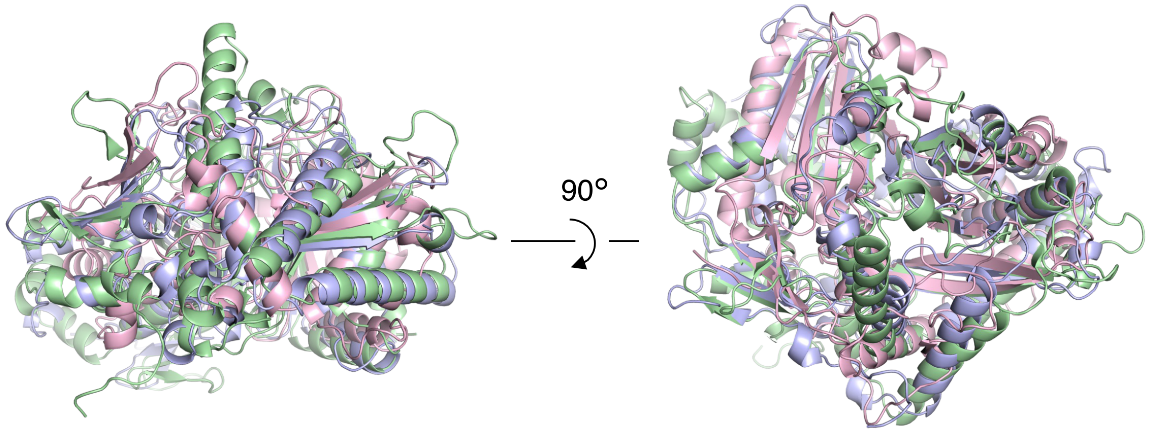

Supplementary Figure 4. Structural superimposition of PtMAC2p (green) TRI3 (light blue; PDB ID: 3fp0), and TRI01 (pink; PDB ID: 3b2s).

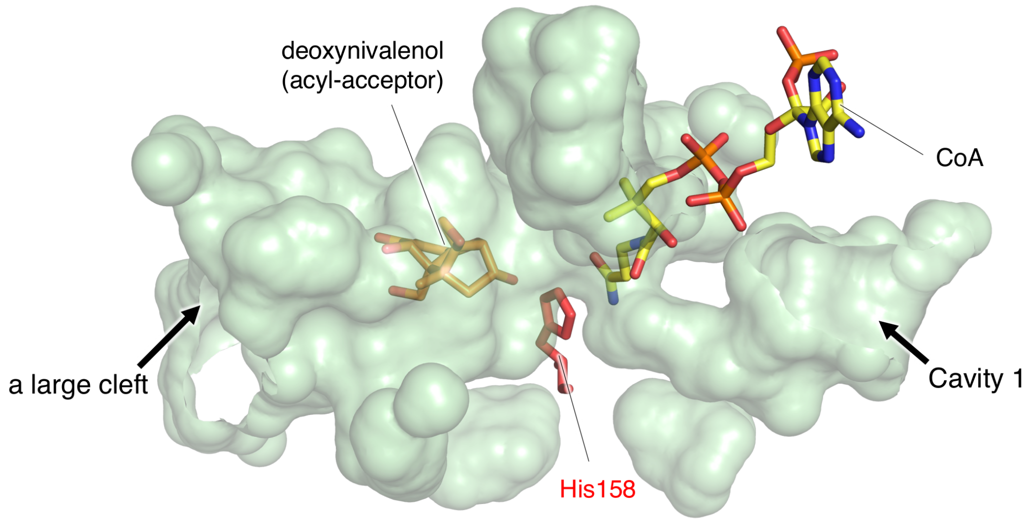

Supplementary Figure 5. The cavity of PtMAC2p is depicted by green. “Cavity 1” shows the putative binding site of acyl-group of acyl-CoA in “Binding state 2” in **Fig. 9**.

```
PtMAC2.                1 --MLGDQVWKEVSSNTLRRPLIGVEKMINYAEYYQNGNFQLTIALSLDTNLSADKMQERL  58
U.hordei_CCF52718      1 --MSASQAWKEVSTNLAKRPLIGVEKLLNYAEYYQNGNFQLSIAMIFETNLLPETLRQRF  58
U.maydis_A0A0D1CRD0    1 -MQADKHAWKEVSPNLAQRPLVGVEKLLNYAEYYQNGNFQLSAAIHLETNLTTEKLQQRF  59
M.aphidis_ETS61960     1 MSTDDNQRWSQVSPDLAKRPLVGVEKVLNYTEHYQDGNFQLTIALLFETSIASETLCERF  60
                              .: *.:**.:  :***:****::**:*:**:*****: *: ::*.: .:.: :*:

PtMAC2                59 GLALWTVRGYLPELGTWTVGSSQDSTLDLDHVTFKAIQTVEEAQEWIEDTAILVDEGTTV 118
U.hordei_CCF52718     59 GLALWIVRGFLPELSTWTVGSKQDSSLDLDHATFAAIQTVEEAQKWIDETALLVEDGTTV 118
U.maydis_A0A0D1CRD0   60 GLAVWNVRCLLPEIGTWTVGTTGDAAVDLDNATFTAIQTVEEAQKWIDETAVIVQDGTTA 119
M.aphidis_ETS61960    61 PLALWSVRSQLPELGTWTVTNDQSAELDLDHALWQPIRSLEQAQQWLHDTAVVANDGTTV 120
                          **:* **  ***:.**** .  .: :***:. : .*:::*:**:*:.:**::.::***.

PtMAC2               119 SEMVDLLSNKRIEPVGKQFRAYLVSQPRHGKPALVMNASHTLNGHRMLFQGSAILQALVD 178
U.hordei_CCF52718    119 EQVIDRTSNQRIEPIGKQFRAYLVSNPREGNPAVIINASHVLNGHRMLFQGCAIVQALVD 178
U.maydis_A0A0D1CRD0  120 EELTILTTNHTIEPAGKQFRVYLVTNARQGSPAIIVNASHVLSGHRIAAQLCTIVQALVD 179
M.aphidis_ETS61960   121 QQMVDRLSNRRIEPVGKQFRVYLVCDPLYGAPGLIVNASHVLNGHRALFQGEMIFQALLD 180
                         .::    :*: *** *****.*** :.  * *.:::****.*.***   *   *.***:*

PtMAC2.              179 ARLTALVASSNSSREALEAVFVPEDISRLARKLPQSLNTAYADKFRPDEADVEAGFAKLG 238
U.hordei_CCF52718    179 GRLAALSEANPDPRSALESIFAPEDLSGLLGKLPISLNTAYEDKFKLGEAEIEAGLEKLG 238
U.maydis_A0A0D1CRD0  180 ARLVSLLQAEPDPRAALRSIFVPENLARLIGKLPISLNTAYHKRFNPTEGDLENGFEKLS 239
M.aphidis_ETS61960   181 PRITGTLRQHPDSTSALAAIFKPEELSESLPKLPQSLNTAYADKFQPGASEIAAGFHKVG 240
                          *:..      ..  ** ::* **:::    *** ****** .:*.   .::  *: *:.

PtMAC2               239 ERILNSTQPTLGIPRFSTPSQNPQYTLGSANGQPLTMLNLRRQIGVTEHRMLHRAFKKRG 298
U.hordei_CCF52718    239 ERLSNGAQSTIGIPRFEQPLSKPLYSLGMLNGEPMTMLNLRRQIGASEHRMLHQAYKKRG 298
U.maydis_A0A0D1CRD0  240 ERLANSALPSIGIPRLSSPATNPEYSLGTVNGEAMTIMNLRRTIGSNEYRLLIDAHKKLG 299
M.aphidis_ETS61960   241 EKLANGAQPSIGIPRFQVPSGTPAFSLGMHDGTPMSMLNLRSRIKAADNQSLKRACKKYG 300
                         *:: *.: .::****:. *  .* ::**  :* .::::***  *   : : *  * ** *

PtMAC2               299 SSLPSFTYACIVNSIDRRCKASTSEADEAPGANLVYSAHASRWFPAETFMSRSPVNMAIV 358
U.hordei_CCF52718    299 STLPSFVYACIVNSIDRRFKANTAEEGEIPGAHLVYSAHASRWFPQETFMRRSPVNMGVV 358
U.maydis_A0A0D1CRD0  300 ITVPSVIYACIVNSIDRRCKSNTAQDAETPGANLAYSAHAKRWLPDETFMTRSPVNMAIV 359
M.aphidis_ETS61960   301 ASVPSLVYACIVNSIDRHCGSSTGSDGAALGANLAYSAHASRWMPAETFMERSPVNMAIV 360
                          ::**. **********:  :.*..     **:*.*****.**:* **** ******.:*

PtMAC2               359 PGSGYVAPEELRSKQRGRDLNESELFALAKTIRAKQEQYLESPHIISYTAQVGDDIAAAM 418
U.hordei_CCF52718    359 MASGYIVPEALRSKQRGRKLNEEEIFQLAKAIRAKQEAYLNTPHIISAMAQVGDEVSNLI 418
U.maydis_A0A0D1CRD0  360 LGSAYVSPDELRSKQHGCDLSIDELIELAKTIRAKQDAYLDTPHIISAMEQVGDEVSAMI 419
M.aphidis_ETS61960   361 LGSGYLSPEELHPGQRGSDLGEAGLFALARTIRQKQDDFLDTPHIIGAMPDLGEQVSSQL 420
                          .*.*: *: *:. *:* .*.   :: **::** **: :*::****.   ::*::::  :

PtMAC2               419 AETANKQRQAGTDPYVALSENSPAICPPTLTSQGEVPIKNLYTPEGASFDPKPKQPEYEY 478
U.hordei_CCF52718    419 AETAIKQREARTDPYVALCENSPAICPPTLTSQGDIPIKRLYTADGASFEPKPKQREGEY 478
U.maydis_A0A0D1CRD0  420 ADTAIKQRQAGTDPHVALFENSPAICPPTLTSQGDIAFKRLYTAQGGSWDPEPAVAAGEY 479
M.aphidis_ETS61960   421 AEAAERQREAGTDWRLALSENSPAVCPPTLTSQGVIPVKRFYTAQGASNKLQLGQPTEEY 480
                         *::* :**:* **  :** *****:********* :..*.:**.:*.* . :      **

PtMAC2               479 IYFGEGLLGGRTTDASVCFALWSLAGILTLQAHFDSRFFDAQVIDTILDDVVLQLRRSAA 538
U.hordei_CCF52718    479 IYFMRGILGGRTTDASVCFAMWTFSGILTLQAHFDSRFFDAKLIDTILDDVVSQLRYSAT 538
U.maydis_A0A0D1CRD0  480 VYIGHSWNSGRTTDASVCFALVGFSGELRLTSYFDSRFFDAKLIESILDDVLSNLRMIAT 539
M.aphidis_ETS61960   481 LEFVDICSGGRTTDASVCFAMFTHAGALTLQAHFDSHFFDAKLVRNILDDVVSQLRAAAA 540
                         : :     .***********:   :* * * ::***:****::: .*****: :**  *:

PtMAC2               539 SAVDEAPEAKL- 549
U.hordei_CCF52718    539 SAVEQRAEAKL- 549
U.maydis_A0A0D1CRD0  540 TVPVDAPEAKL- 550
M.aphidis_ETS61960   541 SASLTSLDQAKL 552
                         :.     :
```

Supplementary Figure 6. Multiple sequence alignment of MAC2p. Mac2p amino acid sequences from *P. tsukubaensis*, *U. hordei* (NCBI accession No., CCF52718), *U. maydis* (A0A0D1CRD0), and *M. aphidis* (ETS61960) were used for allignment. Hydrophobic residues at a large cleft of PtMAC2p are highlighted by blue. Residues corresponding to Arg240 of PtMAC2p are highlighted by orange.
